# Risks in Adolescent Adjustment by Internet Exposure: Evidence From PISA

**DOI:** 10.3389/fpsyg.2021.763759

**Published:** 2021-10-12

**Authors:** Beatriz Ortega-Ruipérez, Almudena Castellanos Sánchez, Beatriz Marcano

**Affiliations:** Educational Technology Department, Faculty of Education, Universidad Internacional de La Rioja, Logroño, Spain

**Keywords:** adolescence, abuse technologies, academic achievement, social adaptation, emotional development

## Abstract

Problematic use and abuse of the Internet has manifested new risks among adolescents that affect academic, social, and emotional adjustment. In the academic domain, the role of Internet use on school performance and learning is studied, including the level of competence as a factor in this domain. On the social level, we measure how Internet use affects school climate and problems related to bullying. On the emotional aspect, the relationship between Internet use and satisfaction and positive feelings is studied, including wellbeing as a construct part of this domain. To find these relationships, data obtained from the Program for International Student Assessment survey, 2018 edition are used. Structural equation modeling was used to determine the most significant relationships between the aspects studied and Internet use. Internet use includes four independent variables: two on abuse (time of use on a weekday outside of class and on a weekend) and two on problematic use (perception about forgetting time and perceived discomfort if the Internet does not work). The results answer three research questions: (1) how the constructs created relate to the four independent variables on Internet usage, (2) how the observed variables respond to the latent constructs, and (3) how all these constructs are related to each other. These results highlight the need to teach young people to use the Internet in a useful and healthy way, as a preventive measure, and help professionals who detect technology abuse to act with effective measures to prevent the psychological consequences, working on the academic, social, and emotional aspects that have shown the greatest relationship. The problematic Internet use is a complex problem and it is impossible and imprudent to relate it to isolated factors: It is necessary to consider different factors to better understand the problem.

## Introduction

Internet use and abuse can lead to Internet addiction in approximately 10 to 20% of users (Akar, 2017; Milková and Ambrožová, 2018). In addition, there are certain risks of Internet use related to problematic Internet use, such as cyberbullying, cyber dating abuse, online grooming, or sexting (Machimbarrena et al., 2018). The most frequent motivation for adolescents to use the Internet is the search for gratification (Cebollero et al., 2021), which in the case of boys is found in entertainment and in the case of girls through evasion. This would explain why boys tend to spend more time on playing social videogames and girls on social media (Twenge and Martin, 2020), resources where the search for social gratification can lead to the development of problematic Internet use.

Most studies on problematic Internet use and abuse focus on the study of individual factors (Anderson et al., 2016), and although there are also quite a few studies on the influence of social factors, individual factors seem to have greater influence (Fumero et al., 2018), such as, for example, low effortful control (Pace et al., 2018). In a study by Chen (2018), in the specific case of Smartphones, it is shown that people with low self-esteem and low social skills are more likely to develop an addiction if they desire the approval of others and a sense of belonging to a group, i.e., the search for gratifications described by Cebollero et al. (2021).

To evaluate all these factors in adolescents, the Programme for International Student Assessment (PISA), promoted by the Organization for Economic Co-operation and Development (OECD), collects and analyzes hundreds of variables in thousands of 15-year-old students around the world. The latest published report shows relationships between some of these factors, grouped in pairs, such as the influence of Internet usage time on wellbeing or the relationship between bullying and school climate (OECD, 2019a). With all these data available, a comprehensive study of which factors have a stronger relationship with Internet use and abuse is essential. For this purpose, individual factors, both academic and emotional, must be considered on the one hand, and social factors on the other.

In academic domain, Internet use should be assessed in terms of learning and performance. On the one hand, moderate and appropriate Internet use can be perceived as usefulness for learning (Huang et al., 2019, 2020). To have an appropriate behavior toward the use of the Internet and to prevent its abuse, it is claimed the need to teach adolescents technological literacy to enhance socio-emotional learning, as well as self-regulation strategies (Schilhab, 2017).

Among adolescents, informal learning with the Internet is motivated by their needs and the influence of their peers (Pereira et al., 2019). Most students feel that learning through YouTube is easier than in class, and many would like to include this tool as another classroom resource (Sulaimanu et al., 2019). In this sense, there are areas where videos can be a great resource because it is easier to understand abstract concepts or concepts that are difficult to understand without visual support. For example, science videos are often used for content enjoyment with an informational use of YouTube (Rosenthal, 2017).

In addition, for informal learning to occur, certain characteristics must be present. For example, this learning occurs more often from browser-based tools than from social media, and computers are used rather than mobile technologies (Lai and Smith, 2017). On the other hand, the use of the Internet for lifelong learning can be much more beneficial if it is approached with a community and social structure than individually (Eynon and Malmberg, 2021), as it can be for language learning in community forums (Clothey, 2017; Liu et al., 2020). With these studies, it is possible to glimpse the benefits that the use of the Internet can offer to learning, if it is approached in an appropriate way.

Performance and learning outcomes can be enhanced by using digital learning (Lin et al., 2017). A well-studied case is the use of virtual laboratories for science, technology, and engineering (Potkonjak et al., 2016). The use of the Internet is also beneficial, and if used in a non-problematic way, it is a good predictor of academic success (Maniaci et al., 2021). In addition, students who use interactive activities and use different tools in a balanced way have higher academic performance than those who only search for information (Torres-Díaz et al., 2016). The use of more tools according to the purpose refers to a high technological literacy, so this is related to greater performance.

However, abuse of the Internet, or problematic use, can have the opposite effect and be detrimental in the academic domain, especially in performance. At a general level, there are contradictory results that do not allow a generalization on how the Internet affects academic performance. In a study with PISA 2015 data, Hu et al. (2018) obtained that the use of information and communication technologies (ICT) at school was positively associated with academic performance, while the use of ICT at home was negatively associated with performance. However, in another study conducted by Rozgonjuk et al. (2021) found that mathematics performance declined the longer the time spent on the Internet, both in and out of school, suggesting that performance may depend more on the time spent on the Internet rather than on where it is used.

However, if other factors, such as emotional factors, are considered when analyzing this relationship, the results are even stronger. On the one hand, the negative effect on performance may be due to low motivation to study, given the effect that problematic Internet use can produce in terms of loneliness, which directly affects motivation to use learning strategies (Truzoli et al., 2020). On the other hand, another mechanism affecting academic performance could be low cognitive-behavioral control, which is related to the stress that excessive use of social media can generate (Masood et al., 2020). Finally, the influence of media multitasking self-efficacy on learning performance in personal learning environments has been seen if students perceive problems in attention and in their learning self-regulation strategies (Wu, 2017).

A strong relationship has also been found between academic performance and bullying, a very serious and quite widespread social problem in society. Bullying victimization is associated with poor academic performance and problems with school attendance (Gardella et al., 2017), also affecting study time and academic engagement (Yu and Zhao, 2021). Students who experience bullying may have worse performance in mathematics, reading, and science (Zhao and Yu, 2020).

At this point, it is necessary to highlight the mediating role of emotional intelligence between academic performance and bullying (Martínez-Martínez et al., 2020). In a study conducted by these authors, students suffering cyberbullying showed low emotional intelligence but did not always show low academic performance. Among all the students who suffered bullying, students with low emotional intelligence had the lowest performance, so that emotional intelligence seems to play an important role in the relationship between these variables. In addition, emotional intelligence plays a similar role for good emotional adjustment in adolescents who experience bullying (Cañas et al., 2020a), so this type of intelligence can be crucial to mitigate the effects of the Internet on poor academic, social, and emotional adjustment.

Internet abuse or its problematic use has a strong relationship with bullying, and especially cyberbullying. According to the results of PISA 2018 (OECD, 2019a), adolescents who spend more time on the Internet are more exposed to bullying, which suggests that in many cases it is cyberbullying, although it has not been measured as such in the study. Cyberbullying is a type of bullying through digital media (Clevenger et al., 2018).

Although an adolescent is more likely to become a victim of cyberbullying than a bully (Çevik et al., 2021), adolescents who are traditionally bullies and who spend more hours on the Internet are more likely to become cyberbullies (Brighi et al., 2019). Furthermore, as stated by Louw and Winter (2011), the characteristics of the internet, a private and unsupervised environment, may be conducive to students who desire acceptance and have a high need to belong, becoming cyberbullies, even if they are not traditionally bullies.

On the other hand, and in addition to the above, bullying is related to problems in social integration and online socialization, lack of school belonging, and loneliness (Yu and Zhao, 2021). So all these results are in line with what we have seen so far and underline the importance of creating a positive and safe school environment, which is known as school climate.

School climate has some predictive value for bullying, specifically the dimensions of academic self-esteem, teacher support, and feeling of affiliation (Ortega-Barón et al., 2016). The sense of approval and belonging to a group is a risk for people with low self-esteem to develop an Internet addiction (Chen, 2018), so having a good school climate can improve self-esteem and decrease the likelihood of developing such addiction. Therefore, at the social level, it is important to consider the school climate as a possible factor related to Internet abuse.

In a study conducted by Zhai et al. (2020), they found a negative association between the perception of school climate and problematic Internet use, mediated by the sense of belonging to the school and depressive symptoms, that is, the perception of a negative school climate reduced the sense of belonging to the school, thus generating depressive symptoms, and increasing problematic Internet use. Meanwhile, Li et al. (2020) obtained results in line with the previous ones, obtaining a negative association between an Internet use disorder and relationships between teachers and students, and relationships between schoolmates.

Regarding the emotional domain, the relationship between Internet use and wellbeing has been studied. On the one hand, the development of digital skills increases the perception of wellbeing among adolescents (Donoso et al., 2020) and leisure Internet use also has a positive association with wellbeing (Lifshitz et al., 2018). On the other hand, time spent on the Internet has a negative but non-significant relationship with wellbeing compared to other variables unrelated to technology (Orben and Przybylski, 2019). This idea is in line with other studies that relate Internet use and wellbeing through the mediation of psychological variables, abilities, and cultural beliefs, without finding a direct relationship between wellbeing and Internet use (Castellacci and Tveito, 2018). The influence of intrapersonal factors, such as low self-esteem, are greater than interpersonal factors on the risk of adolescents with higher Internet use to develop an addiction (Servidio et al., 2019).

As part of wellbeing, life satisfaction is a well-studied variable. No significant relationships have been found between the use of social networks and life satisfaction (Orben et al., 2019), although wellbeing does decrease if the use of social media is problematic (Marino et al., 2017). However, the relationship between higher smartphone and Internet use and lower wellbeing in adolescents has been shown (Twenge et al., 2018), especially when problematic Internet use leads to lower life satisfaction (Lachmann et al., 2018).

Moreover, within the emotional domain, the study of emotions and emotional regulation are of special interest to know how they are related to Internet abuse. The lack of emotional regulation is key to understanding the relationship between childhood trauma and Internet addiction (Evren et al., 2019). In cases of cyberbullying, where there is a clear problematic use of the Internet, both bullies and bullied show this lack of emotional regulation, in addition to more damaged emotional profiles (Cañas et al., 2020b).

In a study in adolescents carried out by Jensen et al. (2019), they found no significant relationship between an increased risk of mental health problems with the days that those adolescents spend more hours on the Internet. Furthermore, George et al. (2018) found that on the days with higher use of technology, there was an increase in symptoms of behavioral problems. So, it cannot be confirmed whether more sporadic Internet use influences mental health problems. However, in studies in which people with widespread problematic Internet and smartphone use are studied, problems of impulsivity, anxiety, and depression have been found (Cimadevilla et al., 2019; Grant et al., 2019).

Regarding the relationship between adolescents’ emotions and the time of Internet use, the PISA 2018 study (OECD, 2019a) shows a clear association between higher negative feelings miserable and sad and higher Internet use, while feelings scared and afraid are slightly lower among heavy users [more than 40 hours(h)/week(w)] than among high users (30–39h/w), although the trend is upward: the higher the use, the higher the negative feelings. As for the positive feelings: happy, lively, proud, joyful, and cheerful, there is a downward trend. That is, the more hours they spend on the Internet, the less they experience these feelings. There is a slight exception with low Internet users (0–9h/w) who experience less of these feelings than moderate Internet users.

This study aims to determine the relationship of all the variables described (academic: performance and learning; social: bullying and school climate; and emotional: wellbeing and emotions) with the use and abuse of the Internet to determine which factors may have a greater influence and which may have a lesser influence.

To create this theoretical model, the starting point was reflection on the dimensions corresponding to each factor to be studied as: academic, social, and emotional. The resulting dimensions had to be composed of several study variables that adequately responded to the corresponding dimension and factor. For this purpose, the indices created by PISA 2018 were reviewed. Most of the study variables were taken directly from these indices, specifically from the Scale indices, which are variables constructed by scaling multiple items (OECD, 2019a). To construct these indices, PISA selected related questions from a broader set, which was based on theoretical considerations and previous research (OECD, 2019b), that is, each index part from a conceptual framework studied in depth. In the case of wellbeing, it was decided to construct an additional variable from items corresponding to life satisfaction that were not included in any of these scales.

The purpose is to better understand how problematic Internet use affects the development of a poor adjustment in any of these factors. This knowledge will serve to detect problems in time attending to the problematic use and abuse of the Internet, and thus be able to design interventions that promote a healthy and fruitful use of it, preventing adjustment problems in these factors.

The questions guiding the research fall into three broad groups: (1) how the constructs created relate to the four independent variables on Internet usage, (2) how the observed variables respond to the latent constructs, and (3) how all these constructs are related to each other.

## Materials and Methods

### Participants

Adolescents from 51 countries participant in PISA, in 2018. In total, there have been 612,004 students aged 15years old who have participated in this program during the course 2017/2018. Of these, 292,946 participants did not answer any of the questions used for the analysis. Therefore, the data used in this study pertain to 319,058 participants, who answered all the questions.

### Research Questions and Measures

To answer the research questions, a structural equation modeling (SEM) is used, a multivariate analysis technique to contrast models that propose causal relationships between variables (Ruiz et al., 2010), which arise to make regression models more flexible, and have a confirmatory rather than an exploratory function (Escobedo et al., 2016). This analysis is more appropriate than a confirmatory factor analysis because SEM expands the possibility of relationships between latent variables and encompasses a measurement model and a structural model (Schreiber et al., 2006). The purpose of SEM is to validate the theory that describes the relationships between variables with empirical data, that is, to confirm a theoretical model based on real information.

This study aims to confirm the relationship between the use of the Internet, including four independent variables (IV): (1) time spent in Internet during a typical weekday (Time_weekday), (2) time spent in Internet during a typical weekend (Time_weekend), (3) perception of forgetting time when using digital devices (Perception_time), and (4) perceived discomfort if no Internet connection is possible (Perceived_discomfort), and different academic, social, and emotional factors.

The measurement model starts from the construction of the dimensions or factors (related to academic, social, and emotional), while the structural model refers to the analysis of the effects between factors and with the Internet use variables, independent variables.

The latent constructs correspond to the factors created and are composed of different observed variables:

- *Academic dimension* is composed of the performance and learning factors, which include the variables: Learning goals (MASTGOAL), Motivation to master tasks (WORKMAST), Enjoyment of reading (JOYREAD), and Value of school (ATTLNACT). For a more detailed explanation of how each of these and other variables was measured, see OECD (2019a). On the other hand, to adequately measure the performance construct, related to the academic dimension, another construct called competence has been included.
- *Competence construct* is composed of the observed variables: Global competence (GLOBAL), Math competencies (MATH), Read competencies (READ), and Science competencies (SCIENCE), which are the averaged results of the items corresponding to each of the tests performed in PISA to measure the level of students in these areas. The results had to be averaged manually because the program used does not do it automatically.
- *Social dimension* is composed of the climate and bullying factors, which include the observed variables: Disciplinary climate (DISCLIMA), Fear of failure (GFOFAIL), and Exposure to bullying (BEINGBULLIED).
- *Emotional dimension* is composed of the wellbeing and feelings factors, which include the variables: Meaning in life (EUDMO), Sense of belonging (BELONG), and Positive feelings (SWBP). On the other hand, to adequately measure the wellbeing construct, related to the emotional dimension, another construct called specifically wellbeing has been included.
- *Wellbeing construct* is composed of 10 observed variables: from Satisf_1 until Satisf_10, which are the 10 items about wellbeing in PISA questionnaire.

The questions guiding the research fall into three broad groups: (1) how the constructs created relate to the four independent variables on Internet usage, (2) how the observed variables respond to the latent constructs, and (3) how all these constructs are related to each other.

In the first question, we start from the general hypothesis that all factors will be related to problematic use and abuse of the Internet, although some factors will be more related than others. In the second question, all the observed variables are expected to respond to the latent constructs, since they have been selected based on the theoretical model proposed in PISA. In the third question, no specific hypothesis is established, since the objective is to explore the possible relationships between constructs in order to gain knowledge about their behavior.

In the structural model, latent constructs can play the role of both independent and dependent variables, causes, and effects of other variables. In addition, this model includes factor loadings, relationships between constructs and error terms for proper interpretation. The model is assessed from factor loadings (measurement model) and regression (structural model) to point out the effects between variables.

On the other hand, different fit indices are analyzed to determine the quality of the model, both global fit and incremental fit. Global fit indices directly measure the ability of the defined model to reproduce the observed data by comparing the estimated variance-covariance matrix with the empirical one. If the difference is equal to zero, a perfect fit is obtained. Incremental fit indices evaluate the model fit by comparing it to an alternative baseline model, so it is a relative fit. This alternative model is usually a null model, which assumes that there is no correlation between the observed variables.

### Procedure and Data Analysis

For quality data collection in all countries participating in the study, PISA provides a document of technical standards and guidelines that is based on consistency, accuracy, generalizability and timelines for test implementation, data management and national standards to ensure cross-national, cross-cultural, and linguistic validity (OECD, 2015).

The PISA assessment framework uses objective tests that allow to know the level of competence in the different domains studied (reading, mathematics, and science), with questions that cover different processes and contexts to achieve an objective assessment. On the other hand, a questionnaire composed of scales is used to assess school-level constructs and non-cognitive or metacognitive constructs. The perception of learning, the social perception of school climate and bullying, and the emotional perception of emotions, satisfaction, and wellbeing correspond to subjective constructs that are assessed through this questionnaire (OECD, 2019b).

In this study, the analysis strategy consisted of three main phases. The first phase had included find out the average scores of the competencies were calculated from the 10 plausible values. With this, we were obtained the four variables related to the competence construct, belonging to the performance factor of the academic dimension. Secondly, missing values were analyzed. Most of the variables could be used because there was no major impact of missing values that could affect the results. However, global competence has more than 50% of the cases without data and has been discarded for the analysis. This provided the definitive variables for the study.

In the second phase, a first model was estimated that assumes that the IVs have effects on all the dependent variables, i.e., it analyzes the effects of the IV on each of the indexes in the database, without including the latent factors. With this first analysis, it was found that there were no significant relationships in general, and the model used in the study was developed, which includes the construction of the five dependent variables from the indexes and relates the IVs to these factors.

In the third and final phase, the model was improved, resulting in a definitive model that allows a much better adjustment of the relationships between the variables studied. To this end, non-significant effects (regression coefficients) were eliminated, and modification indexes were analyzed to check which possible effects could improve the model.

For data analysis, four main indices have been used for the overall assessment of the models, two of them are global fit indices and two are incremental fit indices.

The global fit indices used are Root Mean Square Error of Approximation (RMSEA) and Standardized Root Mean Square Residual (SRMR). The RMSEA reports how well the model fits the reference population. Lower values show better fit, and it is considered acceptable if this index is between 0.05 and 0.08. For its part, the SRMR summarizes the differences between the observed and estimated variance-covariance matrix, based on the study of the residuals. Although there are no fixed values established as cutoff points, finding a value above 0.1 indicates a problem of fit.

The incremental fit indexes used are Normed Fit index (NFI) and Comparative Fit Index (CFI). The NFI is the ratio between the chi-square values of the estimated model and the null model and varies between 0 and 1. To be considered an acceptable fit, the value should be above 0.9. The CFI is an improved version of the NFI, as it is less sensitive to the complexity of models with many parameters. Likewise, 0.9 is established as the cutoff point to consider a good fit.

## Results

**Figure 1:**
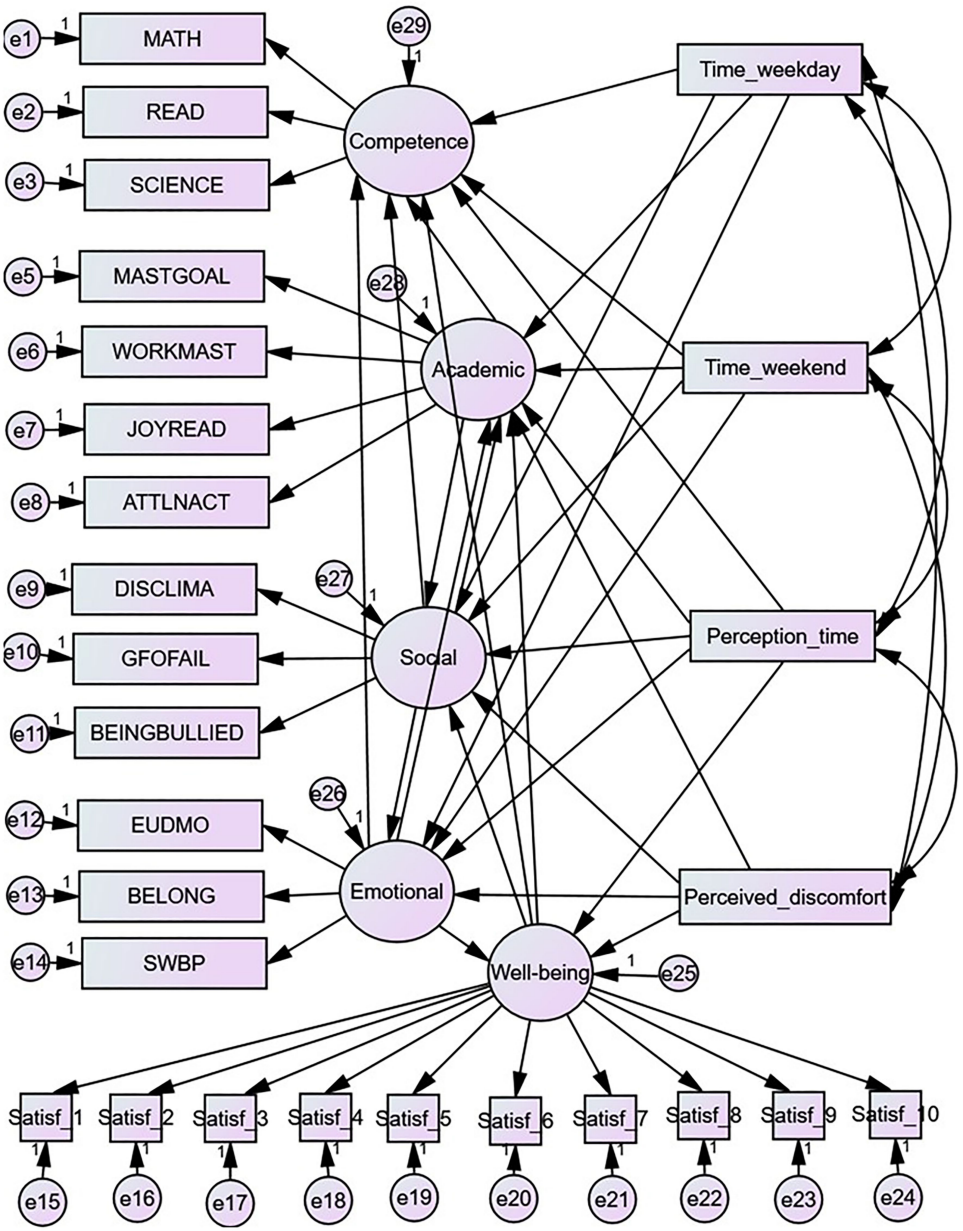
Model and relationship between observed variables and latent constructs.

The model used includes the effects of the IVs related to Internet use and among the five latent factors, that is, it studies the role of cause and effect of all the variables in the model (Figure 1). Based on this proposal, three models have been estimated as: The initial model does not include the effects among the latent factors. The intermediate model includes these effects but eliminates the non-significant effects of the initial model. To establish the effects between latent variables, I have used the modification indexes that indicate the improvement in the quality of the model if this effect is included, but it must also be justified with theory. The final model eliminates all non-significant effects and, as in the previous one, the intensity of the effect, not only the significance, must be checked.

The fit indices show a higher result for the models that include effects between latent factors (intermediate model and final model). The three models achieve acceptable values of the measured indices, becoming very good in the models that include the effects between factors (Table 1).

**Table 1:** Fit indices for the three models.

Model	NPAR	CMIN	DF	*p*	NFI	CFI	RMSEA	SRMR
Initial	76	809772.016	302	0	0.871	0.871	0.066	0.0698
Intermediate	86	616072.486	292	0	0.902	0.902	0.059	0.0424
Final	83	616100.928	295	0	0.902	0.902	0.059	0.0424

The global fit indices, RMSEA obtains a low score, which shows a good fit. Similarly, SRMR shows a low score and away from 0.1. Regarding the incremental fit indices, both NFI and CFI show values above 0.9, so we can consider that the intermediate model and the model have a good fit.

Tables 2–5 include effects from latent factors to independent variables in the final model, and these tables show the regression coefficients for each factor. These first results make it possible to answer the first research question of the study: (1) How the constructs created relate to the four independent variables on Internet usage.

**Table 2:** Results (regression coefficients and significant effects) for the first independent variable: time spent in Internet during a typical weekday (Time_weekday).

Parameter	Regression coefficient	E.T.	*p*
Academic	−0.063	0.002	0.009
Competence	0.112	0.004	0.007
Social	−0.113	0.005	0.015
Emotional	−0.025	0.002	0.009
Wellbeing (non-significant)	0	0	–

**Table 3:** Results (regression coefficients and significant effects) for the second independent variable: time spent in Internet during a typical weekend (Time_weekend).

Parameter	Regression coefficient	E.T.	*p*
Academic (non-significant)	0	0	–
Competence	0.024	0.003	0.009
Social	0.055	0.004	0.013
Emotional	−0.019	0.002	0.005
Wellbeing (non-significant)	0	0	–

**Table 4:** Results (regression coefficients and significant effects) for the third independent variable: perception of forgetting time when using digital devices (Perception_time).

Parameter	Regression coefficient	E.T.	*p*
Academic	0.072	0.005	0.005
Competence	0.075	0.003	0.007
Social	−0.065	0.003	0.006
Emotional	−0.028	0.002	0.009
Wellbeing	0.007	0.002	0.018

**Table 5:** Results (regression coefficients and significant effects) for the fourth independent variable: perceived discomfort if no Internet connection is possible (Perceived_discomfort).

Parameter	Regression coefficient	E.T.	*p*
Academic	0.043	0.007	0.013
Competence (non-significant)	0	0	–
Social	−0.129	0.002	0.011
Emotional	0.018	0.002	0.023
Wellbeing	0.012	0.002	0.015

For the analysis, non-significant effects (*p*<0.05) in previous models were eliminated for the final model analysis. The only non-significant factor for time spent on the Internet during a weekday was wellbeing (Table 2). On the other hand, if the values are significant, it means that the value of the coefficient is the same as that which would be obtained in the population, even if the observed effects are small.

This is the case of the academic and emotional factors, whose coefficients are less than 0.1, the effects are small, but they are applicable to the entire population. Both the academic and emotional factors have an inverse relationship with daily Internet use, that is, the higher the Internet use, it means lower scores on these factors. Regarding the effects that most explain the time spent on the Internet during a typical weekday, it can be seen how competence is positively related, while the social factor is negatively related.

In the case of the variable Internet use on weekends, Table 3 shows that both the academic factor and the wellbeing factor are not significant. The other factors (competence, social, and emotional) are significant, but their effect is very small (less than 0.1). In this case, we see that the competence factor maintains a direct relationship and the emotional factor a slight inverse relationship, as occurred with the time of use during the week. However, in contrast to weekday use, the hours of Internet use on weekends have a positive relationship with the social factor.

For the variable perception of forgetting time when using digital devices, Table 4 shows significant relationships with all factors. However, its effect is very small, being less than 0.1 in all cases. Interestingly, the academic and competence factors have a direct relationship, meaning that the higher the perception of forgetting time, better are the learning outcomes. While the social and emotional factors have an inverse relationship, the higher the perception of forgetting time, worse are the results in the social and emotional domains. The wellbeing factor has a regression coefficient that is too small to be considered (less than 0.01).

Finally, in the case of the variable perceived discomfort if no Internet connection is possible, the competence factor is not significant. The social factor has the greatest effect of all, with an inverse relationship, in other words, the greater the perceived discomfort, the lower the score on the social factor. The remaining factors have a very small effect (less than 0.1), but positive in all cases, i.e., a direct relationship. Being a variable related to discomfort, it is curious that it does not have an inverse relationship with a greater effect on the emotional and wellbeing factors.

On the other hand, in Tables 6–10, the regression results of each of the latent factors are collected to answer the other two research questions of the study: (2) how the observed variables respond to the latent constructs and (3) how these constructs are related to each other.

**Table 6:** Results (regression coefficients and significant effects) for academic factor.

Parameter	Regression coefficient	E.T.	*p*
Competence	0.57	0.012	0.01
Social	−0.67	0.037	0.006
MASTGOAL	0.668	0.001	0.006
WORKMAST	0.631	0.002	0.009
JOYREAD	0.28	0.002	0.011
ATTLNACT	0.386	0.002	0.006

**Table 7:** Results (regression coefficients and significant effects) for competence construct.

Parameter	Regression coefficient	E.T.	*p*
MATH	0.933	0	0.007
READ	0.945	0	0.008
SCIENCE	0.979	0	0.005

**Table 8:** Results (regression coefficients and significant effects) for social factor.

Parameter	Regression coefficient	E.T.	*p*
Academic	0.354	0.058	0.008
Competence	0.716	0.013	0.009
DISCLIMA	0.326	0.003	0.012
GFOFAIL	−0.28	0.004	0.012
BEINGBULLIED	−0.432	0.002	0.006

**Table 9:** Results (regression coefficients and significant effects) for emotional factor.

Parameter	Regression coefficient	E.T.	*p*
Academic	0.452	0.038	0.012
Competence	−0.924	0.018	0.018
Social	1.12	0.026	0.009
Wellbeing (non-significant)	0	0	–
EUDMO	0.584	0.002	0.009
SWBP	0.545	0.002	0.012
BELONG	0.48	0.002	0.007

**Table 10:** Results (regression coefficients and significant effects) for wellbeing construct.

Parameter	Regression coefficient	E.T.	*p*
Academic	−0.012	0.002	0.006
Competence	0.043	0.003	0.005
Social	−0.019	0.003	0.003
Emotional	0.279	0.002	0.012
Satisf_1	0.653	0.003	0.025
Satisf_2	0.623	0.003	0.012
Satisf_3	0.602	0.004	0.018
Satisf_4	0.668	0.003	0.019
Satisf_5	0.668	0.003	0.021
Satisf_6	0.722	0.003	0.01
Satisf_7	0.628	0.003	0.013
Satisf_8	0.713	0.002	0.025
Satisf_9	0.664	0.003	0.016
Satisf_10	0.694	0.003	0.009

To answer how the observed variables respond to the latent constructs, we must look at the variables related to the academic factor (Table 6). While Learning goals (MASTGOAL) and Motivation to master tasks (WORKMAST) have a high effect on the factor (>0.6), Enjoyment of reading (JOYREAD) and Value of school (ATTLNACT) have a much lower effect (0.28 and 0.38). Moreover, as explained in the research method, the Competence construct is considered part of the academic factor, and this can be confirmed given the relatively high effect it has shown (0.57).

Additionally, to answer how the academic factor is explained by other factors, we obtained an inverse relationship with a strong effect with the social factor, meaning that a higher academic score is associated with a lower social score.

In the case of the competence construct, Table 7 shows that the three observed variables that respond to this construct (mathematics, reading, and science scores) explain the competence construct with a very high effect (>0.9). Since it is considered part of the academic domain, the relationship with the rest of the factors has not been measured.

Table 8 shows the observed variables that respond to the social factor. In this case, a medium-low effect (absolute values between 0.2 and 0.5) can be seen in the explanation of this factor due to the three variables. While Disciplinary climate (DISCLIMA) has a direct relationship with the social factor, that is, a higher score on the social factor, greater score on the school climate; Fear of failure (GFOFAIL) and Exposure to bullying (BEINGBULLIED) have an inverse relationship, with Exposure to bullying showing a stronger effect explaining the social factor. This means that if the fear of failure is lower and the exposure to bullying is lower, the social factor score will be higher.

In addition, Table 8 shows how other factors are related to the social factor. In this case, it is interesting to note that the academic factor explains the social factor directly with a medium-low effect (0.35), but if we focus on the competence construct, it explains the social factor with a high effect (>0.7).

For the emotional factor, Table 9 shows how the observed variables respond with a medium effect (between 0.48 and 0.58), a direct relationship on this factor. Specifically, from highest to lowest effect, we find as: Meaning in life (EUDMO), Sense of belonging (BELONG), and Positive feelings (SWBP).

Also, Table 9 shows that the emotional factor is not significantly explained by wellbeing, although wellbeing is explained by the emotional factor (Table 10). In addition, we see how the emotional factor is explained with a significant effect by academic and social factors. The academic factor is the smallest, which explains the emotional factor with a medium effect (0.45). Meanwhile, competence construct explains the emotional factor inversely and with a very strong effect. This means that those who have a higher level of competence will have a lower score in the emotional factor, which would explain why students with better grades may have more emotional problems.

In relation to this result, a very high effect of the social factor in explaining the emotional factor is observed. A possible hypothesis that could arise from the interaction between these last results is that students with higher grades (higher competence) are not always socially accepted, which would have a negative impact on their emotional side. Undoubtedly, these results need further investigation in subsequent studies.

Finally, Table 10 shows how the observed variables that respond to the wellbeing construct all have a medium-high effect in explaining wellbeing (between 0.6 and 0.72), given that they are all specific items that have been chosen to evaluate wellbeing.

In the case of how this construct relates to others, as mentioned above, it has a direct relationship with a medium effect with the emotional factor. Initially, in the method, the wellbeing construct has been included as part of the emotional factor, so this relationship is confirmed. In addition, with the other factors, the wellbeing construct has a very low effect (less than 0.1), while the emotional factor had obtained quite high effects. This supports the theoretical proposal that wellbeing is part of the emotional factor, and the rest of the factors are explained by the emotional factor and not by the wellbeing construct. Future studies should confirm these relationships.

## Discussion

The results found allow us to establish interpretations that improve our knowledge of how different factors related to the academic, social, and emotional domains are affected by the development of problematic Internet use. To discuss the results, research questions 1 and 2 will be interpreted first, to know how the academic, competence, social, emotional, and well-being factors respond to the independent variables related to Internet use, and to be able to explain which observed variables best explain each of these factors. Question 3 is then interpreted to find out what significant relationships exist between the different latent factors.

### Problematic Internet Use in Academic, Social, and Emotional Domains

First, with respect to time spent using the Internet, we must differentiate between Internet use during a typical weekday and Internet use during a typical weekend. It is notable that wellbeing does not have a significant relationship with the time spent using the Internet, neither on weekdays nor on weekends. In this sense, the results support the idea that the relationship between both variables is not direct but affects other aspects, such as cultural (Castellacci and Tveito, 2018) or intrapersonal variables (Servidio et al., 2019), variables not related to technology (Orben and Przybylski, 2019), contrary to the results of other authors who do certify the relationship between time of Internet use and wellbeing (Lifshitz et al., 2018; Donoso et al., 2020).

The use of the Internet during the week is explained to a greater extent by the social factor, meaning that students who spend more time on the Internet may have more problems in the social sphere. According to the results, students who spend more time on the Internet during the week may have greater exposure to bullying, worse perception of school climate, and/or feel more fear of failure.

Weekday Internet use is also largely explained by the competence construct: Students who use the Internet longer during the week have higher scores in math, reading, and science. These results are in line with the studies of Huang et al. (2019, 2020), if their use is not problematic, because ICT use can improve performance and outcomes (Lin and Chen, 2017).

However, students who use the Internet more during the week have greater problems with the academic domain in general, i.e., learning. Among these problems are that these students are less able to set their learning goals and are lower motivated to manage their homework, and that they value school less highly. Also, although with a smaller effect according to Internet use, they have less fun with reading.

At this point, it is remarkable that, while adolescents who use the Internet more during the week obtain better academic results (competence), they have more problems with learning and the academic domain, in general. Further inquiry is required to adequately deepen this finding, as it may depend on the type of tools they tend to use, some more suitable than others for learning, as Lai and Smith (2017) expose.

The emotional factor also has a significant, although small, relationship with weekday Internet use: the higher the weekday Internet use, the lower the emotional satisfaction. Emotional satisfaction is given by perceived meaning in life, sense of belonging, and experiencing positive feelings. This could be in line with the results of Chen (2018) who saw that people with low self-esteem are more likely to develop addiction to their smartphones, in search of improving their personal satisfaction through increasing their sense of belonging to a virtual group.

Regarding Internet use on weekends, there is also a significant relationship, although with a much smaller effect, between the construct of competence and Internet use: the greater the Internet use on a typical weekend, higher levels of competence. In line with the findings of Maniaci et al. (2021), Internet use is a good predictor of academic success, provided it is not used in a problematic way. The emotional factor also explains Internet use on weekends: more Internet use, decreased emotional satisfaction. Both the results regarding competence and the emotional domain are in line with the observed results for weekday Internet use.

However, those who use the Internet for more hours on weekends have higher scores in the social factor, as opposed to those who use it during the week. This could be explained by a differential use of the Internet: While during the week it would be used more for academic tasks, on weekends it would be used as a leisure resource. This hypothesis would also be supported by the fact that no significant relationship was found between Internet use on weekends and the academic factor. In view of these results, we do not share the findings of Hu et al. (2018) that greater use of ICT at home is associated with poor performance, nor the results of Rozgonjuk et al. (2021) students who spend more time on the Internet outside of school have lower math scores.

Time spent on the Internet can be considered as a problem related to Internet abuse. However, problematic Internet use can also occur even if only a few hours are spent on the Internet. For this reason, it was considered important to include other variables related to the perceptions of adolescents when they use it inappropriately. For this reason, the other two independent variables have been chosen to provide more clarity to the interpretation of the results.

An important issue in terms of problematic Internet use is the perception of forgetting time when using digital devices. In this case, although the effect of the factors is small, in all cases it is significant. In the case of the academic factor, and their related construct, competence, a direct relationship has been obtained with the perception of forgetting time. This means that, when this perception occurs more frequently, these factors are higher. Therefore, better students are more likely to develop this problem. At this point, the development of self-regulation strategies could help, as pointed out by Schilhab (2017). Meanwhile, the social and emotional factors have an inverse relationship with the perception of forgetting time. In this case, this problematic perception of losing track of time occurs more often when the scores in the social and emotional domains are lower.

The other important question for detecting problematic Internet use is perceived discomfort if no Internet connection is possible. In this case, the level of competence does not have a significant relationship with this problem, although the academic factor is significant and has a considerable effect. The higher the perceived discomfort, the higher the scores on this factor. These results seem to be related to the results obtained in the problem of perception of forgetting time while surfing the Internet. Adolescents with more problems in the use of the Internet seem to have a greater predisposition to learning if we do not consider the rest of factors. However, it is also important to consider other factors that have a greater effect in explaining this problem. In the case of perceived discomfort if no Internet connection is possible, the social factor has a greater intensity in explaining this problem, with an inverse relationship. This means that adolescents with higher perceived discomfort have lower scores in the social domain, in line with previous studies (Li et al., 2020; Zhai et al., 2020).

In this problem, emotional and wellbeing factors also have a significant relationship, although with a much smaller effect than social and academic factors. In these two cases, the relationships are direct: those who experience higher perceived discomfort, higher emotional satisfaction, and/or wellbeing. It is quite possible that the satisfaction and/or wellbeing experienced are related to the possibilities offered to them by the Internet, such as the need for social interaction according to Chen (2018). In these cases, it would be appropriate to explore further how the problem is related to these factors and to include an intermediate explanatory variable to control the effect of experiencing satisfaction due to Internet use.

### Relationship Between the Latent Factors

Regarding the third research question about how the relationship between the latent factors is, it has been obtained that the academic factor is more explained by the social factor, although in an inverse way, followed by the competence construct (which is part of the academic factor according to the theoretical proposal). These results are in line with proposals that there is a strong relationship between bullying and achievement and commitment to study (Gardella et al., 2017; Zhao and Yu, 2020; Yu and Zhao, 2021). At this point, it is worth recalling the need for training on emotional intelligence to mediate these effects (Martínez-Martínez et al., 2020; Cañas et al., 2020a).

For its part, the social factor is largely explained by the competence construct, but not as part of the academic factor, which has shown a much lower relationship with the social factor. The higher the level of competence, a positive and safe school environment is generated, increasing the sense of belonging to the school, which could explain the relationship with the social factor (Yu and Zhao, 2021).

On the other hand, the emotional factor is highly explained by the social factor, whose relationship can be explained by the high need for social approval experienced by many adolescents, which undoubtedly affects their emotional satisfaction and wellbeing, through obtaining gratifications, according to Cebollero et al. (2021). The emotional factor is also largely explained by the level of competence, although in an inverse manner. And, as has occurred with the social factor, the effect is much smaller if the academic factor is considered as a whole.

Finally, an unexpected result was that the wellbeing construct does not explain the emotional factor, since the relationship is not significant. However, the emotional factor does explain wellbeing, so the relationship to confirm the theoretical model between both constructs should be investigated in future studies.

As a conclusion to this study, it is confirmed that problematic Internet use is a complex problem, so there may be multiple relationships between factors related to academic, social, and emotional factors that are influenced by the way in which this resource is used. It is impossible and imprudent to relate the development of problems in the use of the Internet to isolated factors, and therefore, it is necessary to consider different factors to better understand the relationships between all of them and address the problem in a comprehensive manner.

The main practical implications of the results obtained are related to the aspects to which we should pay more attention when a problem is detected in a specific area. In other words, by obtaining stronger relationships between certain variables, we can interpret that if there is a problem in a given variable, the variables that have obtained stronger relationships will be the ones most likely to develop new problems.

Table 11 shows a summary of the main practical implications of the study. First, the problems with the Internet that can generate greater problems in specific factors are shown and, subsequently, each of the factors studied is shown, together with the dimensions that have obtained a greater relationship and, therefore, can develop the next problems.

**Table 11:** Summary of relevant practical implications.

If a problem is detected in …	Be especially careful with problems in …
Time spent during a typical weekday	Social factor
Perceived discomfort if no internet connection is possible	Social factor
Academic factor	Ambitious learning goals and motivation to master task
Competence factor	All domains: math, read, science
Social factor	Exposure to bullying
Emotional factor	Perception about meaning in life, and ability to sense positive feelings
Wellbeing factor	Emotional factor

Even so, it is important to re-emphasize that the relationships between all the factors and dimensions must always be considered, since all of them show relationships, whether major or minor, so that secondary problems may arise in any of them.

Therefore, this study brings to this field of research the importance of considering the influence of problematic Internet use and abuse on different factors in a related way, and not as isolated factors.

One limitation of the study may be that it worked with so many variables and so many data, which may have influenced the results of the analyses. To mitigate these drawbacks, as far as possible, new hypotheses and research questions have been proposed throughout the discussion of the results, which will serve as future lines of research on problematic Internet use.

Another limitation has been the need to create an additional variable to measure wellbeing, since there is no specific scale among the scale indexes created by PISA. This implies less theoretical support, and less validity, given that no additional tests have been carried out. In this sense, a future line of action would be to test the consistency of the wellbeing variable created, in the same way that the rest of the scale indices have been validated by the PISA program.

## Data Availability Statement

Publicly available datasets were analyzed in this study. This data can be found at: https://webfs.oecd.org/pisa2018/SPSS_STU_QQQ.zip.

## Ethics Statement

Ethical review and approval was not required for the study on human participants in accordance with the local legislation and institutional requirements. Written informed consent to participate in this study was provided by the participants’ legal guardian/next of kin.

## Author Contributions

BO-R conducted the main literature search, drafted the theoretical framework, and wrote the results, discussion and conclusions. ACS and BM reviewed the theoretical framework, contributed complementary sources, reviewed the article, and provided complementary ideas. All authors agreed on the research approach and data analysis.

## Conflict of Interest

The authors declare that the research was conducted in the absence of any commercial or financial relationships that could be construed as a potential conflict of interest.

## Publisher’s Note

All claims expressed in this article are solely those of the authors and do not necessarily represent those of their affiliated organizations, or those of the publisher, the editors and the reviewers. Any product that may be evaluated in this article, or claim that may be made by its manufacturer, is not guaranteed or endorsed by the publisher.
